# Surgical timing, neurological recovery, and survival in spinal metastasis-induced paralysis: a retrospective cohort study with nomogram development

**DOI:** 10.3389/fonc.2026.1901644

**Published:** 2026-09-07

**Authors:** Shuo Gong, Lei Zhou, Changhua Wang

**Affiliations:** Department of Orthopedic and Soft Tissue Surgery, Shandong Cancer Hospital and Institute, Shandong First Medical University, Shandong Academy of Medical Sciences, Jinan, Shandong, China

**Keywords:** neurological recovery, nomogram, paralysis, spinal metastasis, surgical timing, epidural spinal cord compression

## Abstract

**Objective:**

To investigate the association between surgical timing and neurological recovery and survival in patients with complete or incomplete paralysis due to metastatic spinal cord compression, and to develop a nomogram for predicting marked neurological recovery.

**Methods:**

A total of 302 patients who underwent surgical decompression for spinal metastasis-induced paralysis between 2019 and 2023 were retrospectively analyzed. Patients were divided into four groups based on the time from paralysis onset to surgery: <24h, 24–72h, 72h–1w, and >1w. The primary outcome was marked neurological recovery (improvement to AIS D/E). Survival outcomes were also assessed. Multivariable Firth penalized logistic regression and Cox proportional hazards regression were performed. A nomogram was constructed and validated using bootstrap resampling (C-index, calibration curve, ROC curve, and decision curve analysis).

**Results:**

Earlier surgery was strongly associated with better neurological recovery. Compared with the <24h group, the odds ratios for marked recovery were 0.69 (24–72h, p=0.361), 0.34 (72h–1w, p=0.019), and 0.17 (>1w, p<0.001). Delayed surgery was associated with significantly increased mortality risk: hazard ratios were 1.87 (72h–1w, p=0.026) and 4.44 (>1w, p<0.001). Preoperative AIS grade C (OR=6.86, p<0.001), higher ESCC grade (grade 2 vs. 1C: OR=0.14; grade 3 vs. 1C: OR=0.28), and flaccid muscle tone (OR=0.23, p<0.001) were independent predictors of recovery. The nomogram showed excellent discrimination with a bootstrap-corrected C-index of 0.873 and an AUC of 0.888 (95% CI: 0.852–0.925). Decision curve analysis confirmed its clinical utility.

**Conclusion:**

Early surgery (within 1 week, ideally <24h) was associated with better neurological recovery and longer survival in patients with spinal metastasis-induced paralysis. However, given the retrospective design and the absence of key oncological covariates—including performance status, systemic disease burden, and visceral metastases—the survival association should be interpreted with caution and does not imply causality. Preoperative AIS grade C was associated with better recovery, while higher ESCC grade (grade 2 vs. 1C: OR=0.14; grade 3 vs. 1C: OR=0.28) was associated with lower odds of recovery, indicating that patients with less severe cord compression (ESCC grade 1C) had better outcomes. Flaccid muscle tone predicted poorer outcomes. The proposed nomogram provides a preliminary tool for individualized prediction of marked neurological recovery, pending external validation.

## Introduction

Spinal metastases are a frequent complication of advanced cancer, affecting approximately 15–30% of patients with solid tumors (1). Metastatic epidural spinal cord compression (MESCC) represents a particularly severe manifestation, occurring in 5–10% of cancer patients and frequently resulting in irreversible paralysis and substantial impairment of quality of life (2). The most common primary tumors associated with spinal metastases are lung, breast, and prostate cancers (3). Preservation or restoration of ambulatory function is therefore a critical treatment goal, as neurological status is strongly associated with functional independence and quality of life in this patient population (4).

Prompt surgical decompression plays a central role in the management of MESCC. The landmark randomized trial by Patchell et al. demonstrated that direct decompressive surgery followed by postoperative radiotherapy significantly improved postoperative ambulation compared with radiotherapy alone in selected patients with metastatic spinal cord compression (5). It should be noted that this trial established the superiority of combined surgery plus radiotherapy over radiotherapy alone, rather than surgery as a standalone treatment. Since then, advances in surgical techniques, perioperative management, and multidisciplinary cancer care have further expanded the role of surgery in preserving neurological function and improving patient outcomes (3).

Despite the established benefit of surgery, the optimal timing of intervention remains uncertain. Several studies have suggested that earlier surgery is associated with superior neurological recovery. Fürstenberg et al. reported improved neurological outcomes among patients treated within 48 hours of symptom onset. Similarly, Fan et al. found that surgery within 48 hours was associated with better restoration of ambulation in patients with complete motor paralysis, while Chaichana et al. identified a symptom duration of less than 48 hours as an independent predictor of postoperative ambulation (7, 8). Li et al. further demonstrated that surgical intervention within one week could still provide meaningful neurological benefit in patients with complete paralysis (9). However, whether the critical therapeutic window lies within 24 hours, 48 hours, or one week remains unclear.

Moreover, surgical timing alone is unlikely to fully explain postoperative recovery, as neurological outcomes are influenced by multiple patient- and disease-related factors. Preoperative neurological status, as measured by ASIA grade, is widely regarded as the strongest predictor of postoperative recovery (5). The severity of epidural spinal cord compression, graded using the Bilsky Epidural Spinal Cord Compression (ESCC) scale, has also been associated with neurological outcomes and represents an important indicator of spinal cord compromise (10). Experimental studies suggest that prolonged mechanical compression may result in substantial motor neuron loss, with flaccid paralysis potentially reflecting irreversible neuronal injury (11). Nevertheless, the interplay between surgical timing, neurological status, compression severity, and muscle tone has not been systematically investigated. A better understanding of these interactions may facilitate individualized treatment decisions and improve prognostic assessment in patients with MESCC-associated paralysis.

Therefore, the objectives of the present study were to: (1) evaluate the association between surgical timing and neurological recovery and overall survival in a large cohort of patients with spinal metastasis-induced paralysis (AIS grades A-C); (2) identify independent predictors of marked neurological recovery (AIS grades D/E) and overall survival; (3) investigate the interaction among ESCC grade, AIS grade, and surgical timing; and (4) develop and internally validate a nomogram for individualized prediction of neurological recovery.

## Methods

### Study design and patient population

This retrospective study was conducted at the Department of Orthopedic Oncology, Shandong Cancer Hospital (Shandong First Medical University Affiliated Cancer Hospital), Jinan, China. The study protocol was approved by the institutional review board.

A total of 302 patients who underwent surgical decompression for spinal metastasis-induced paralysis between 2019 and 2023 were retrospectively reviewed. Inclusion criteria were: (1) severe motor deficits due to metastatic spinal cord compression, corresponding to AIS grades A, B, or C (hereafter referred to as “paralysis” for brevity, encompassing both complete paralysis [AIS A-B] and severe incomplete motor deficits [AIS C]); (2) MRI evidence of epidural spinal cord compression with ESCC grade 1C, 2, or 3; (3) single-level or contiguous multilevel compression; (4) expected survival > 3 months. Exclusion criteria were: (1) spinal shock, defined as a transient state of spinal cord dysfunction occurring within 24–48 hours of acute spinal cord injury, characterized by complete loss of motor, sensory, and reflex function below the level of injury, including absence of the bulbocavernosus reflex; (2) multiple discrete compressive lesions, defined as two or more non-contiguous levels of epidural spinal cord compression on MRI, separated by at least one uninvolved spinal segment; (3) concomitant brain metastases or cauda equina compression; (4) pre-existing neurological deficits due to comorbidities (e.g., diabetes, stroke); (5) benign tumors; (6) surgery performed > 2 weeks after onset of paralysis; (7) preoperative AIS grade D. Of note, patients with lumbar spine tumors were included only if the lesion was located at or above the conus medullaris (typically L1–L2 level) and resulted in spinal cord compression rather than cauda equina compression. Tumors at L2 or below causing isolated cauda equina syndrome were excluded, as the pathophysiology and recovery potential differ from spinal cord compression. The distinction was made based on MRI findings and clinical presentation, with lesions causing upper motor neuron signs (e.g., hyperreflexia, spasticity, Babinski sign) classified as spinal cord compression, while those causing lower motor neuron signs only (e.g., areflexia, isolated radiculopathy) were classified as cauda equina syndrome and excluded.

### Data collection

Clinical data were extracted from electronic medical records, including demographics (age, sex), clinical presentation (paralysis type, muscle tone), MRI findings (ESCC grade, tumor location, number of spinal levels involved), surgical records (timing from paralysis onset to surgery), pathological findings (primary tumor site), and follow-up data (neurological status, survival status). Follow-up was performed at discharge, every 2 weeks for the first month, every 4 weeks for the next 6 months, and every 3 months thereafter until death or 24 months after surgery.

Muscle tone was assessed preoperatively by the treating spine surgeon within 24 hours prior to surgery and classified as flaccid (hypotonia with absent or diminished deep tendon reflexes) or spastic (hypertonia with hyperactive deep tendon reflexes, clonus, or positive Babinski sign). The distinction between flaccid paralysis and spinal shock was made based on clinical criteria: patients presenting within 24–48 hours of acute injury onset with global areflexia and absent bulbocavernosus reflex were classified as having spinal shock and were excluded from the study. Flaccid paralysis in the absence of spinal shock features (i.e., present bulbocavernosus reflex, onset >48 hours, or selective hyporeflexia) was considered to reflect lower motor neuron involvement or severe spinal cord injury and was included. This classification was performed by two investigators (S.G. and L.Z.), with disagreements resolved by consensus review of clinical documentation.

### Surgical procedure and timing

All operations were performed by the senior author. The surgical approach (posterior decompression and fixation, anterior decompression and fixation, or combined anterior-posterior approach) was determined based on tumor location and extent of cord compression. The timing of surgery was defined as the interval from the documented onset of complete or incomplete paralysis to surgery and categorized into four groups: <24 hours, 24–72 hours, 72 hours to 1 week, and >1 week. Paralysis onset was determined retrospectively based on medical records and was defined as the time point at which the patient or family first reported inability to move the lower extremities (complete paralysis) or significant motor weakness affecting ambulation (incomplete paralysis, corresponding to AIS grade C or worse). In cases where the exact onset time was uncertain, the earliest documented time of motor deficit was used. This definition was applied consistently across all patients by two investigators (S.G. and L.Z.), with discrepancies resolved by consensus.

Rationale for timing categories: Prior studies have established that surgery within 48 hours is associated with improved neurological outcomes (6–8). However, patients with metastatic spinal cord compression are typically in advanced stages of malignancy with significant comorbidities, rendering surgical intervention inherently high-risk. In clinical practice, comprehensive preoperative optimization—including correction of coagulopathy, cardiopulmonary assessment, nutritional support, and multidisciplinary team (MDT) evaluation—is often necessary to ensure patient safety. Additionally, adequate time is required for patient counseling regarding the substantial perioperative risks, which may influence decision-making and consent. Consequently, achieving surgery within 24 or even 48 hours represents an ideal rather than a consistently attainable goal in real-world practice; in our cohort, only 25.8% of patients underwent surgery within 24 hours and 56.6% within 72 hours. Given these clinical realities, the present study aimed to explore a broader, more pragmatic therapeutic window by categorizing patients into four timing groups (<24h, 24–72h, 72h–1w, and >1w). This design allowed us to evaluate whether meaningful neurological benefit could still be achieved with surgery performed within one week, providing clinically actionable information for settings where ultra-early intervention is not feasible. To facilitate comparison with prior literature using the traditional 48-hour threshold, additional sensitivity analyses (<48h vs. ≥48h, <72h vs. ≥72h, and continuous time modeling) were performed and are presented in the **Supplementary Materials** (**Supplementary Tables S-A0–S-B2**).

### Neurological and survival outcomes

Neurological function was assessed preoperatively and at standardized postoperative time points (at discharge, and at 1, 3, and 6 months, and every 3 months thereafter when feasible) using the AIS. The primary outcome was marked neurological recovery, defined as improvement to AIS grade D or E at the last available follow-up. All patients survived at least 2 months postoperatively and received at least one postoperative neurological assessment at hospital discharge; patients who died before subsequent follow-up assessments were analyzed using the last-observation-carried-forward approach. Sensitivity analyses excluding patients with early mortality (≤2 months and ≤3 months) were performed to assess the robustness of findings (**Supplementary Table**). Overall survival was defined as the time from surgery to death or last follow-up (censored at 24 months).

### Radiological assessment

ESCC was graded according to the Bilsky ESCC scale (10): grade 1C (epidural disease with thecal sac deformation and spinal cord abutment but without cord compression), grade 2 (spinal cord compression with cerebrospinal fluid space still visible around the cord), and grade 3 (spinal cord compression with no visible cerebrospinal fluid space around the cord). All MRI scans were independently reviewed by two spine surgeons; discrepancies were resolved by consensus with reference to a neuroradiologist when necessary.

### Statistical analysis

Continuous variables were presented as mean ± standard deviation or median with interquartile range, and categorical variables as frequencies with percentages. Baseline characteristics were compared among the four surgical timing groups using the chi-square test for categorical variables and the Mann-Whitney U test or Kruskal-Wallis test for continuous variables, as appropriate.

To identify predictors of marked neurological recovery, a Firth penalized maximum likelihood logistic regression model was used to address complete separation issues. The model included surgical timing, age, sex, preoperative AIS grade, ESCC grade, and muscle tone. Results were reported as odds ratios with 95% confidence intervals. Predictor selection was based on clinical relevance and prior literature, with all prespecified variables entered into the multivariable model simultaneously. No stepwise selection was performed to avoid overfitting and to maintain clinical interpretability. The bootstrap validation (1000 resamples) included the entire model-building process, including predictor estimation and model fitting, to obtain optimism-corrected performance estimates.

For survival analysis, the Kaplan-Meier method was used to estimate overall survival, and differences among groups were assessed using the log-rank test. Multivariable Cox proportional hazards regression was performed to identify independent predictors of survival, including the same covariates as in the logistic model. Hazard ratios with 95% confidence intervals were reported.

A nomogram was constructed based on the final multivariable logistic regression model to predict the probability of marked neurological recovery. Model performance was assessed using bootstrap resampling (1000 repetitions) to calculate the bias-corrected C-index. Calibration curves were plotted to evaluate agreement between predicted and observed probabilities. The mean absolute error was calculated to quantify calibration accuracy. Receiver operating characteristic curve analysis was performed to calculate the area under the curve. Decision curve analysis was conducted to evaluate the clinical utility of the nomogram for identifying patients likely to achieve marked neurological recovery. In this analysis, the “treat all” strategy represented the assumption that all patients would undergo surgery, while the “treat none” strategy represented the assumption that no patients would undergo surgery. The net benefit of the nomogram was compared against these two reference strategies across a range of threshold probabilities. It should be noted that, as all patients in this cohort underwent surgery, the “treat all” and “treat none” reference lines do not represent clinically actionable alternatives in this setting; the DCA is therefore presented as an illustration of the nomogram’s potential for risk stratification within a surgical cohort, rather than as a tool for defining treatment thresholds.

All statistical analyses were performed using R software (version 4.5.3, R Foundation for Statistical Computing, Vienna, Austria) with the logistf, survival, rms, rmda, and pROC packages. A two-sided p-value < 0.05 was considered statistically significant.

## Results

### Patient characteristics

**Figure 1:**
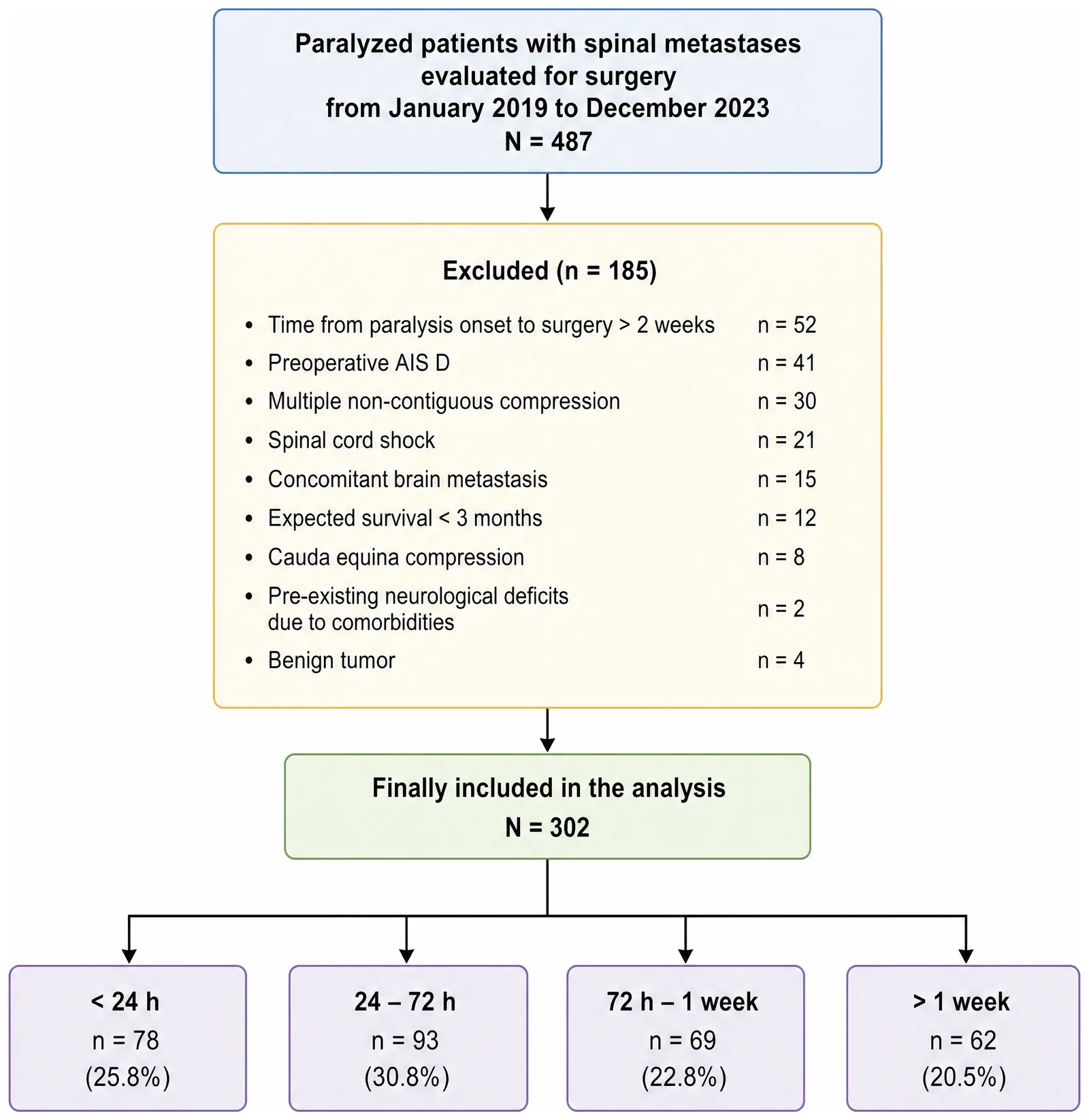
Patient selection flow diagram. Patients with spinal metastasis-induced paralysis who underwent surgical decompression at Shandong Cancer Hospital between 2019 and 2023 were screened for eligibility. Patients were excluded for the following reasons: spinal shock, multiple discrete non-contiguous compressive lesions, concomitant brain metastases or cauda equina compression, pre-existing neurological deficits due to comorbidities, benign tumor histology, surgery performed more than 2 weeks after paralysis onset, or preoperative AIS grade D. A total of 302 patients met all inclusion criteria and were included in the final analysis. AIS, ASIA Impairment Scale; ESCC, epidural spinal cord compression.

A total of 302 patients with spinal metastasis-induced paralysis were included in this study. The patient selection process is summarized in the study flow diagram (**Figure 1**). The median age was 55 years (mean 55.3 ± 9.8 years, range: 31–79 years), and 186 patients (61.6%) were male, while 116 patients (38.4%) were female (**Table 1**). The median follow-up time was 19 months (IQR: 6–24 months).

**Table 1:** Baseline characteristics by surgical timing group.

Characteristic	Level	<24h (N=78)	24–72h (N=93)	72h–1w (N=69)	>1w (N=62)	Overall (N=302)	p-value
Age (years), median (mean±SD)		55 (54.4 ± 8.9)	55 (55.7 ± 9.4)	54 (54.7 ± 9.8)	56 (56.3 ± 11.3)	55 (55.3 ± 9.8)	
Sex							0.311
	Female	37 (47.4%)	34 (36.6%)	23 (33.3%)	22 (35.5%)	116 (38.4%)	
	Male	41 (52.6%)	59 (63.4%)	46 (66.7%)	40 (64.5%)	186 (61.6%)	
Paralysis type							<0.001
	Complete	15 (19.2%)	29 (31.2%)	34 (49.3%)	35 (56.5%)	113 (37.4%)	
	Incomplete	63 (80.8%)	64 (68.8%)	35 (50.7%)	27 (43.5%)	189 (62.6%)	
ESCC (Bilsky) grade							0.295
	1C	27 (34.6%)	27 (29%)	24 (34.8%)	25 (40.3%)	103 (34.1%)	
	2	21 (26.9%)	37 (39.8%)	23 (33.3%)	13 (21%)	94 (31.1%)	
	3	30 (38.5%)	29 (31.2%)	22 (31.9%)	24 (38.7%)	105 (34.8%)	
Preoperative AIS							<0.001
	A	10 (12.8%)	27 (29%)	32 (46.4%)	33 (53.2%)	102 (33.8%)	
	B	27 (34.6%)	35 (37.6%)	20 (29%)	16 (25.8%)	98 (32.5%)	
	C	41 (52.6%)	31 (33.3%)	17 (24.6%)	13 (21%)	102 (33.8%)	
Muscletone							<0.001
	Flaccid	18 (23.1%)	42 (45.2%)	35 (50.7%)	50 (80.6%)	145 (48%)	
	Spastic	60 (76.9%)	51 (54.8%)	34 (49.3%)	12 (19.4%)	157 (52%)	
Tumor location							0.458
	cervical	8 (10.3%)	2 (2.2%)	6 (8.7%)	4 (6.5%)	20 (6.6%)	
	lumbar	18 (23.1%)	19 (20.4%)	14 (20.3%)	12 (19.4%)	63 (20.9%)	
	thoracic	52 (66.7%)	72 (77.4%)	49 (71%)	46 (74.2%)	219 (72.5%)	
Spinal levels involved							0.493
	Multiple	15 (19.2%)	11 (11.8%)	8 (11.6%)	9 (14.5%)	43 (14.2%)	
	Single	63 (80.8%)	82 (88.2%)	61 (88.4%)	53 (85.5%)	259 (85.8%)	

Values are presented as median (mean ± SD) for age, or n (%) for categorical variables. SD, standard deviation; AIS, American Spinal Injury Association Impairment Scale; ESCC, epidural spinal cord compression.

Bold values indicate statistically significant results (p < 0.05).

The cohort was divided into four groups based on the time from onset of complete or incomplete paralysis to surgery: <24 hours (n=78, 25.8%), 24–72 hours (n=93, 30.8%), 72 hours to 1 week (n=69, 22.8%), and >1 week (n=62, 20.5%).

### Baseline characteristics

Significant differences were observed among the four groups in paralysis type, preoperative AIS grade, and muscle tone (all p < 0.001). Patients in the >1 week group had the highest proportion of complete paralysis (56.5%), AIS A grade (53.2%), and flaccid muscle tone (80.6%), whereas those in the <24h group had the highest proportion of incomplete paralysis (80.8%), AIS C grade (52.6%), and spastic muscle tone (76.9%). Sex (p = 0.311), tumor location (p = 0.458), and spinal levels involved (p = 0.493) were comparable across the four groups (**Table 1**). Among the 103 patients with ESCC grade 1C, the distribution of preoperative AIS grades was: AIS A (n=21, 20.4%), AIS B (n=34, 33.0%), and AIS C (n=48, 46.6%) (**Supplementary Ta****ble S-C1**).

### Primary tumor distribution

The most common primary tumor sites were lung (149 patients, 49.3%), breast (44, 14.6%), liver (28, 9.3%), and esophagus (19, 6.3%). Other tumor types accounted for the remaining 20.5% of cases (**Table 2**).

**Table 2:** Distribution of primary tumor sites.

Primary tumor site	N	Percentage
Lung	149	49.3%
Breast	44	14.6%
Liver	28	9.3%
Esophagus	19	6.3%
Kidney	13	4.3%
Prostate	10	3.3%
Stomach	6	2%
Bladder	4	1.3%
Cervix	3	1%
Colon	3	1%
Melanoma	3	1%
Rectum	3	1%
Soft tissue	3	1%
Thyroid	3	1%
Cardia	2	0.7%
Duodenum	2	0.7%
Mediastinum	2	0.7%
Skin	2	0.7%
Anus	1	0.3%
Lymphoma	1	0.3%
Nasal cavity	1	0.3%
Total	302	100%

Values are presented as n (%).

### Perioperative outcomes

Perioperative complications occurred in 75 patients (24.8%), including cerebrospinal fluid leak (n=45, 14.9%), wound infection (n=30, 9.9%), pneumonia (n=8, 2.6%), deep vein thrombosis (n=5, 1.7%), hardware failure (n=3, 1.0%), and other complications such as pressure ulcers and urinary tract infections (n=6, 2.0%).

### Neurological recovery outcomes

**Figure 2:**
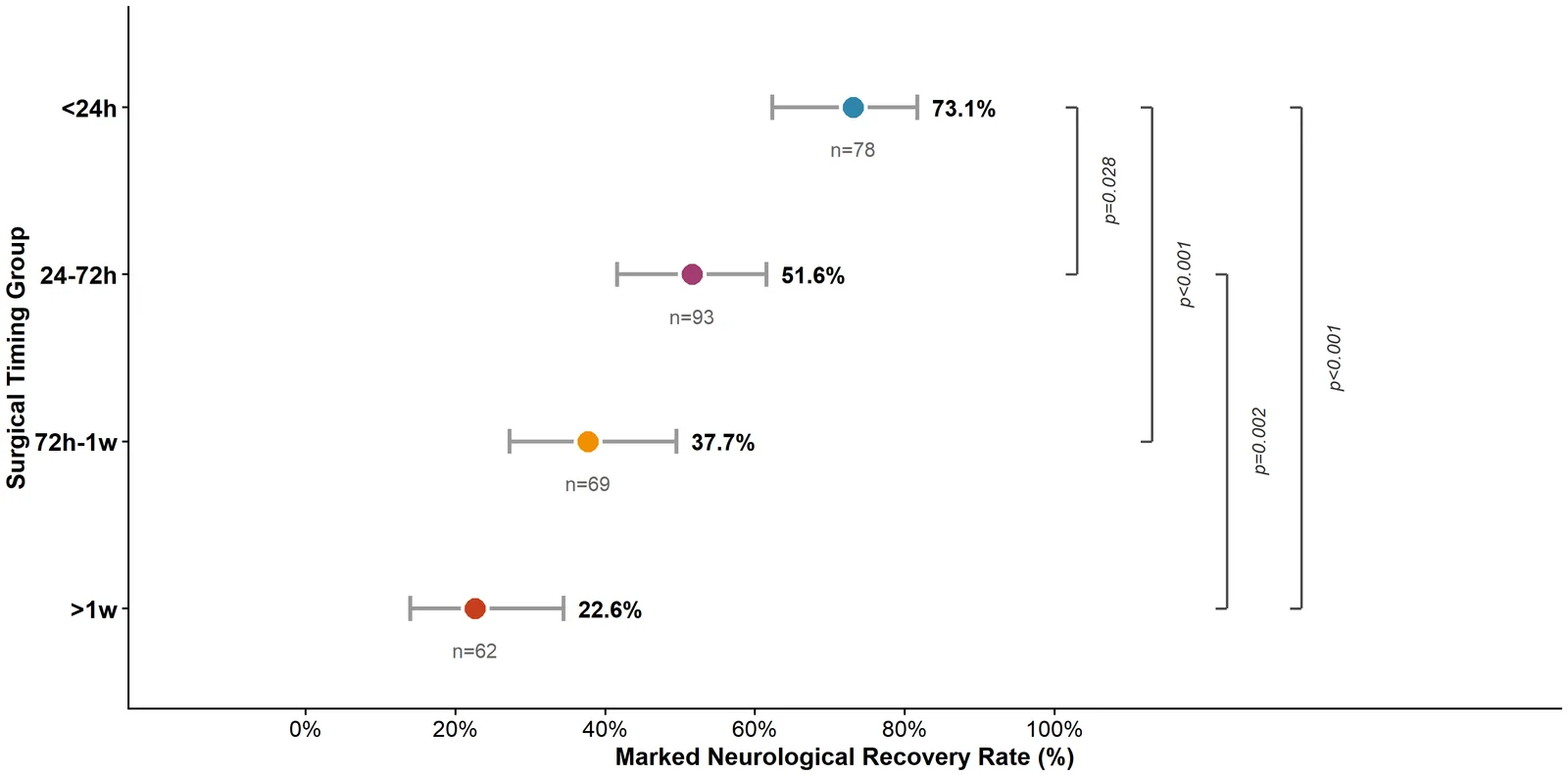
Marked neurological recovery rates by surgical timing group. Points represent recovery rates with 95% Wilson confidence intervals; sample sizes are shown below each point. Pairwise Fisher’s exact tests with Bonferroni correction were performed. Recovery rates decreased progressively with delayed surgery: <24h (73.1%), 24–72h (51.6%), 72h–1w (37.7%), and >1w (22.6%). The difference between <24h and >1w was significant (p < 0.001). CI, confidence interval; h, hours; w, week.

Of the 302 patients, the proportion achieving marked neurological recovery (AIS D/E) decreased progressively with delayed surgery: <24h (73.1%, n=78), 24–72h (51.6%, n=93), 72h–1w (37.7%, n=69), and >1w (22.6%, n=62) (**Figure 2**). Pairwise comparisons using Fisher’s exact test with Bonferroni correction showed significant differences between all adjacent groups (all p < 0.05) (**Figure 2**).

### Multivariable analysis for neurological recovery

**Figure 3:**
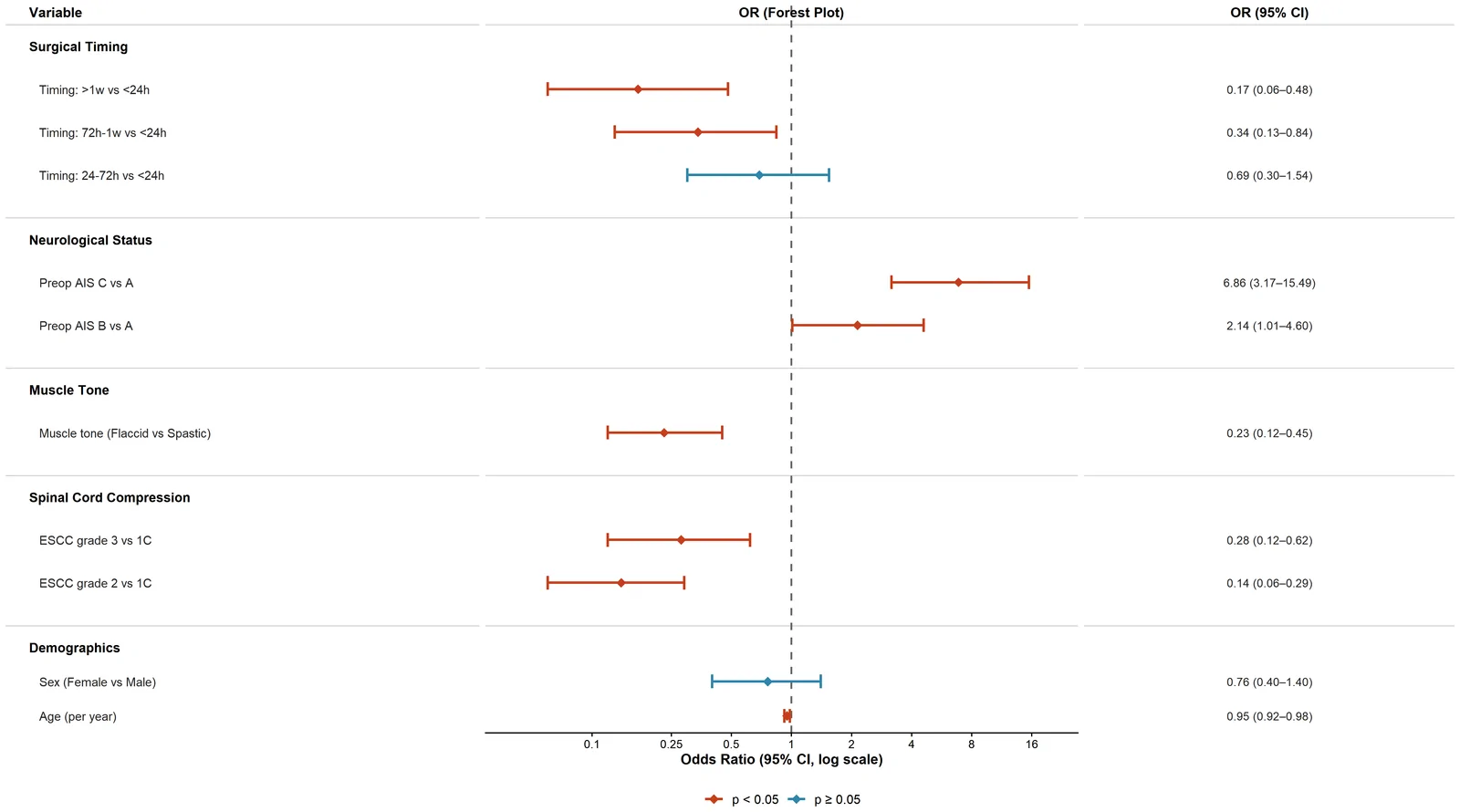
Firth penalized logistic regression for marked neurological recovery (AIS D/E). Forest plot of odds ratios (ORs) with 95% CIs. Variables grouped into five categories. Red circles indicate p < 0.05; blue circles p ≥ 0.05. Earlier surgery (<24h), higher preoperative AIS grade (B/C), spastic muscle tone, and lower ESCC grade (1C) were associated with better recovery. Older age was associated with lower odds of recovery (p = 0.002), while sex was not an independent predictor (p = 0.378). OR, odds ratio; CI, confidence interval; AIS, American Spinal Injury Association Impairment Scale; ESCC, epidural spinal cord compression; h, hours; w, week.

A Firth penalized logistic regression model was constructed to identify independent predictors of marked neurological recovery, adjusting for surgical timing, age, sex, preoperative AIS grade, ESCC grade, and muscle tone (**Table 3**; **Figure 3**).

**Table 3:** Firth penalized logistic regression for marked neurological recovery (AIS D/E).

Variable	OR (95% CI)	p-value
Timing: 24-72h vs <24h	0.69 (0.3-1.54)	0.361
Timing: 72h-1w vs <24h	0.34 (0.13-0.84)	0.019*
Timing: >1w vs <24h	0.17 (0.06-0.48)	<0.001***
Age (per year)	0.95 (0.92-0.98)	0.002**
Sex (Female vs Male)	0.76 (0.4-1.4)	0.378
Preop AIS B vs A	2.14 (1.01-4.6)	0.048*
Preop AIS C vs A	6.86 (3.17-15.49)	<0.001***
ESCC grade 2 vs 1C	0.14 (0.06-0.29)	<0.001***
ESCC grade 3 vs 1C	0.28 (0.12-0.62)	0.002**
Muscle tone (Flaccid vs Spastic)	0.23 (0.12-0.45)	<0.001***

1. OR, odds ratio; CI, confidence interval; AIS, American Spinal Injury Association Impairment Scale; ESCC, epidural spinal cord compression.

2. *p < 0.05; **p < 0.01; ***p < 0.001.

3. Reference groups:Timing <24h, ESCC 1C, Preop AIS A, Muscle tone spastic, Sex male.

Earlier surgery within 1 week was associated with significantly better neurological recovery. Compared with the <24h reference group, the odds ratios (ORs) for marked recovery were 0.69 (95% CI: 0.30–1.54, p = 0.361) for the 24–72h group, 0.34 (95% CI: 0.13–0.84, p = 0.019) for the 72h–1w group, and 0.17 (95% CI: 0.06–0.48, p < 0.001) for the >1w group. Notably, the 24–72h group did not show a statistically significant difference compared to the <24h group (p = 0.361).

Preoperative AIS grade was a strong independent predictor. Compared with AIS A, the OR for AIS B was 2.14 (95% CI: 1.01–4.60, p = 0.048), and the OR for AIS C was 6.86 (95% CI: 3.17–15.49, p < 0.001). Higher ESCC grade was associated with significantly lower odds of marked recovery: ESCC grade 2 vs. 1C (OR = 0.14, 95% CI: 0.06–0.29, p < 0.001) and ESCC grade 3 vs. 1C (OR = 0.28, 95% CI: 0.12–0.62, p = 0.002). Flaccid muscle tone was associated with markedly lower odds of recovery compared with spastic tone (OR = 0.23, 95% CI: 0.12–0.45, p < 0.001). Older age was also associated with lower odds of recovery (OR = 0.95 per year, 95% CI: 0.92–0.98, p = 0.002). Sex was not an independent predictor (p = 0.378) (**Table 3**; **Figure 3**).

### Sensitivity analyses for early mortality

In this cohort, no patients died within 1 month of surgery (minimum postoperative survival: 2 months); therefore, all 302 patients received at least one postoperative neurological assessment at hospital discharge. Two patients (0.7%) died within 2 months and 25 patients (8.3%) died within 3 months of surgery. Sensitivity analyses excluding these early deaths (n = 300 and n = 277, respectively) yielded results consistent with the primary analysis, with surgical timing, preoperative AIS grade, ESCC grade, and muscle tone remaining significant predictors of marked neurological recovery (**Supplementary Table**). These findings support the robustness of the primary results.

### Survival analysis

**Figure 4:**
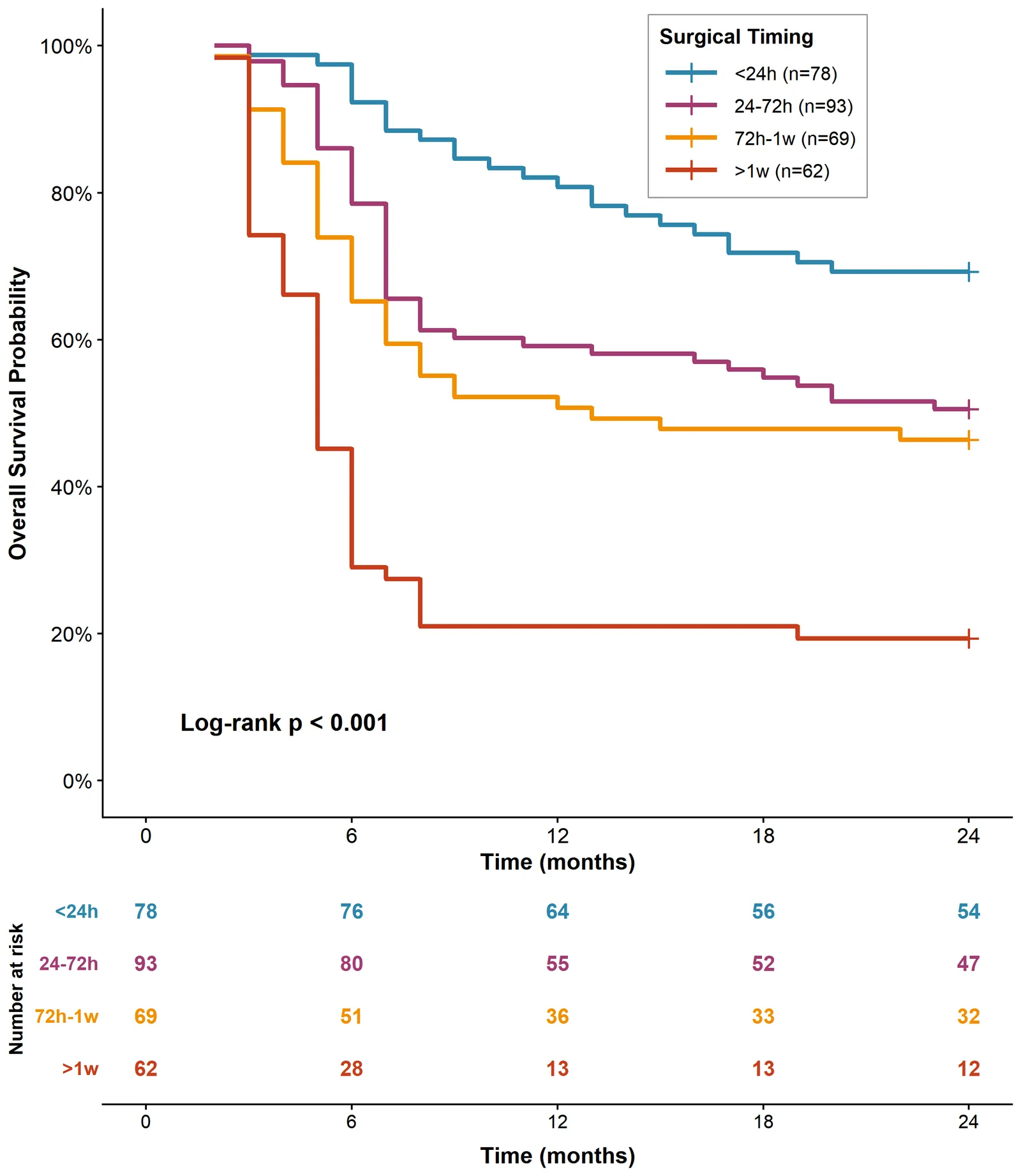
Overall survival curves by surgical timing group. Kaplan–Meier estimates of overall survival over 24 months. Vertical tick marks indicate censored observations; number at risk shown below x-axis. Survival decreased significantly with delayed surgery (log-rank p < 0.001). The <24h group had the highest survival; the >1w group had the lowest. h, hours; w, week.

The overall survival probability decreased significantly with delayed surgery (log-rank p < 0.001) (**Figure 4**). The 24-month survival probabilities were 69.2% (95% CI: 59.7%–80.3%) for the <24h group (blue), 50.5% (95% CI: 41.3%–61.8%) for the 24–72h group (purple), 46.4% (95% CI: 36.0%–59.8%) for the 72h–1w group (yellow), and 19.4% (95% CI: 11.6%–32.2%) for the >1w group (red) (**Figure 4**). The median survival time was not reached for the <24h group, while the >1w group had the shortest median survival (approximately 12 months). The >1w group showed the steepest decline in survival, particularly within the first 6 months, while the <24h group maintained the highest survival probability throughout the 24-month follow-up period. The number at risk at each time point is shown below the x-axis in **Figure 4**.

### Multivariable analysis for overall survival

**Figure 5:**
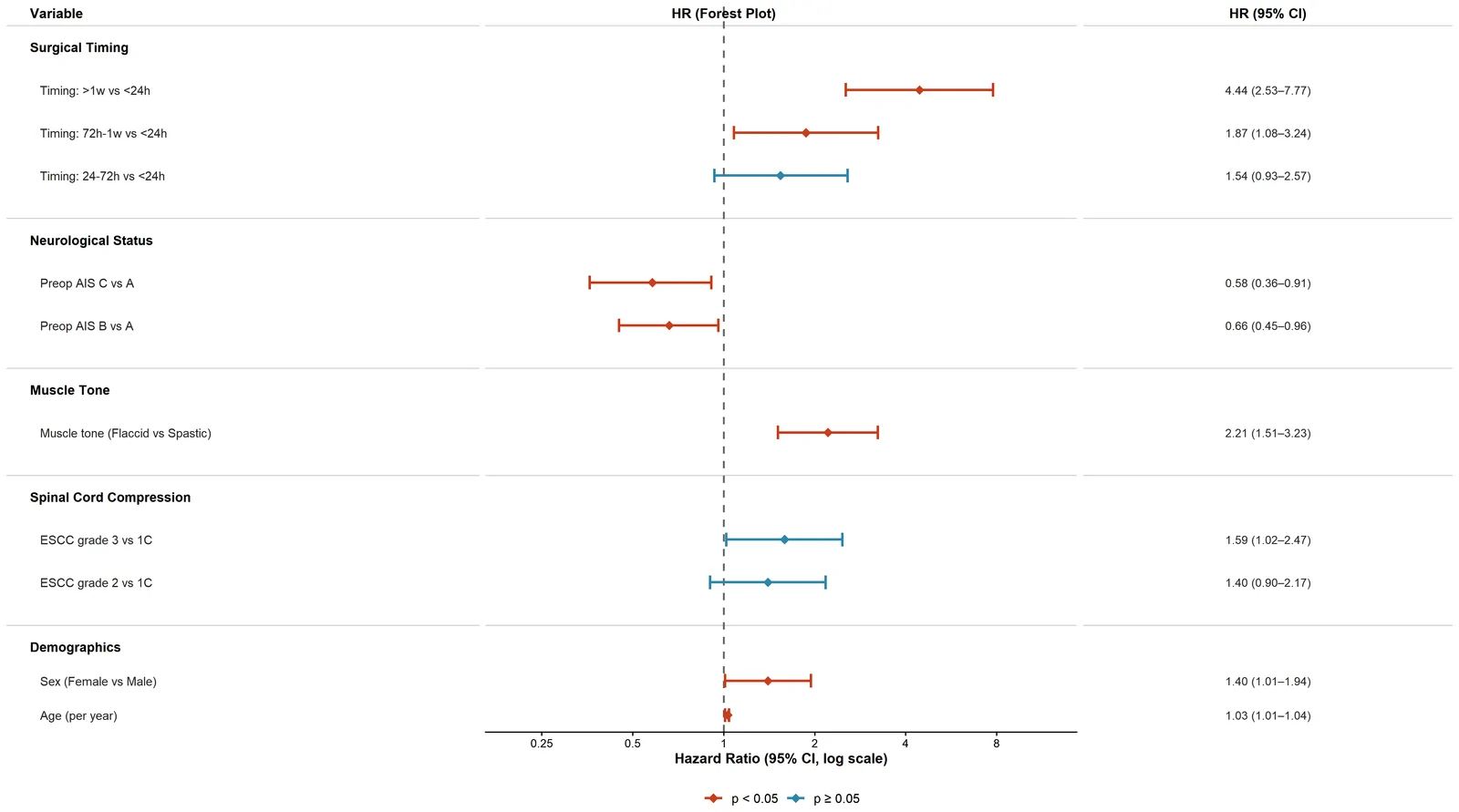
Multivariable Cox regression for overall survival. Forest plot of hazard ratios (HRs) with 95% CIs. Variables grouped into five categories. Red circles indicate p < 0.05; blue circles p ≥ 0.05. Delayed surgery (>72h), flaccid muscle tone, higher ESCC grade (3), female sex, and older age were associated with increased mortality risk; higher preoperative AIS grade (B/C) was associated with reduced risk. HR, hazard ratio; CI, confidence interval; AIS, American Spinal Injury Association Impairment Scale; ESCC, epidural spinal cord compression; h, hours; w, week.

A Cox proportional hazards regression model was constructed to identify independent predictors of overall survival, adjusting for surgical timing, age, sex, preoperative AIS grade, ESCC grade, and muscle tone (**Table 4**; **Figure 5**).

**Table 4:** Multivariable Cox proportional hazards regression for overall survival.

Variable	HR (95% CI)	p-value
Timing: 24-72h vs <24h	1.54 (0.93-2.57)	0.095
Timing: 72h-1w vs <24h	1.87 (1.08-3.24)	0.026*
Timing: >1w vs <24h	4.44 (2.53-7.77)	<0.001***
Age (per year)	1.03 (1.01-1.04)	0.005**
Sex (Female vs Male)	1.4 (1.01-1.94)	0.044*
Preop AIS B vs A	0.66 (0.45-0.96)	0.03*
Preop AIS C vs A	0.58 (0.36-0.91)	0.019*
ESCC grade 2 vs 1C	1.4 (0.9-2.17)	0.135
ESCC grade 3 vs 1C	1.59 (1.02-2.47)	0.039*
Muscle tone (Flaccid vs Spastic)	2.21 (1.51-3.23)	<0.001***

1. HR, hazard ratio; CI, confidence interval; AIS, American Spinal Injury Association Impairment Scale; ESCC, epidural spinal cord compression.

2. Reference groups: Timing <24h, ESCC 1C, Preop AIS A, Muscle tone spastic, Sex male.

Delayed surgery was associated with significantly increased mortality risk. Compared with the <24h reference group, the hazard ratios (HRs) for mortality were 1.54 (95% CI: 0.93–2.57, p = 0.095) for the 24–72h group, 1.87 (95% CI: 1.08–3.24, p = 0.026) for the 72h–1w group, and 4.44 (95% CI: 2.53–7.77, p < 0.001) for the >1w group. Notably, the 24–72h group showed a trend toward increased mortality risk but did not reach statistical significance (p = 0.095).To assess the robustness of these findings, a sensitivity analysis was performed restricting the cohort to patients with ESCC grade 2 or 3 (n = 199, events = 118), excluding the 103 patients with ESCC grade 1C. The surgical timing effect remained directionally consistent and numerically stronger: the hazard ratio for the >1w group was 5.57 (95% CI: 2.91–10.66, p < 0.001), compared with 4.44 in the primary analysis. Results for neurological recovery were similarly consistent (**Supplementary Tables S-D1**, **S-D2**).

Preoperative AIS grade was a protective factor for survival. Compared with AIS A, the HR for AIS B was 0.66 (95% CI: 0.45–0.96, p = 0.030), and the HR for AIS C was 0.58 (95% CI: 0.36–0.91, p = 0.019). Higher ESCC grade was associated with increased mortality risk: ESCC grade 3 vs. 1C (HR = 1.59, 95% CI: 1.02–2.47, p = 0.039), while ESCC grade 2 did not reach statistical significance (HR = 1.40, 95% CI: 0.90–2.17, p = 0.135). Flaccid muscle tone was associated with markedly increased mortality risk compared with spastic tone (HR = 2.21, 95% CI: 1.51–3.23, p < 0.001). Older age (HR = 1.03 per year, 95% CI: 1.01–1.04, p = 0.005) and female sex (HR = 1.40, 95% CI: 1.01–1.94, p = 0.044) were also independent predictors of increased mortality risk (**Table 4**; **Figure 5**).

### Interaction analysis

**Figure 6:**
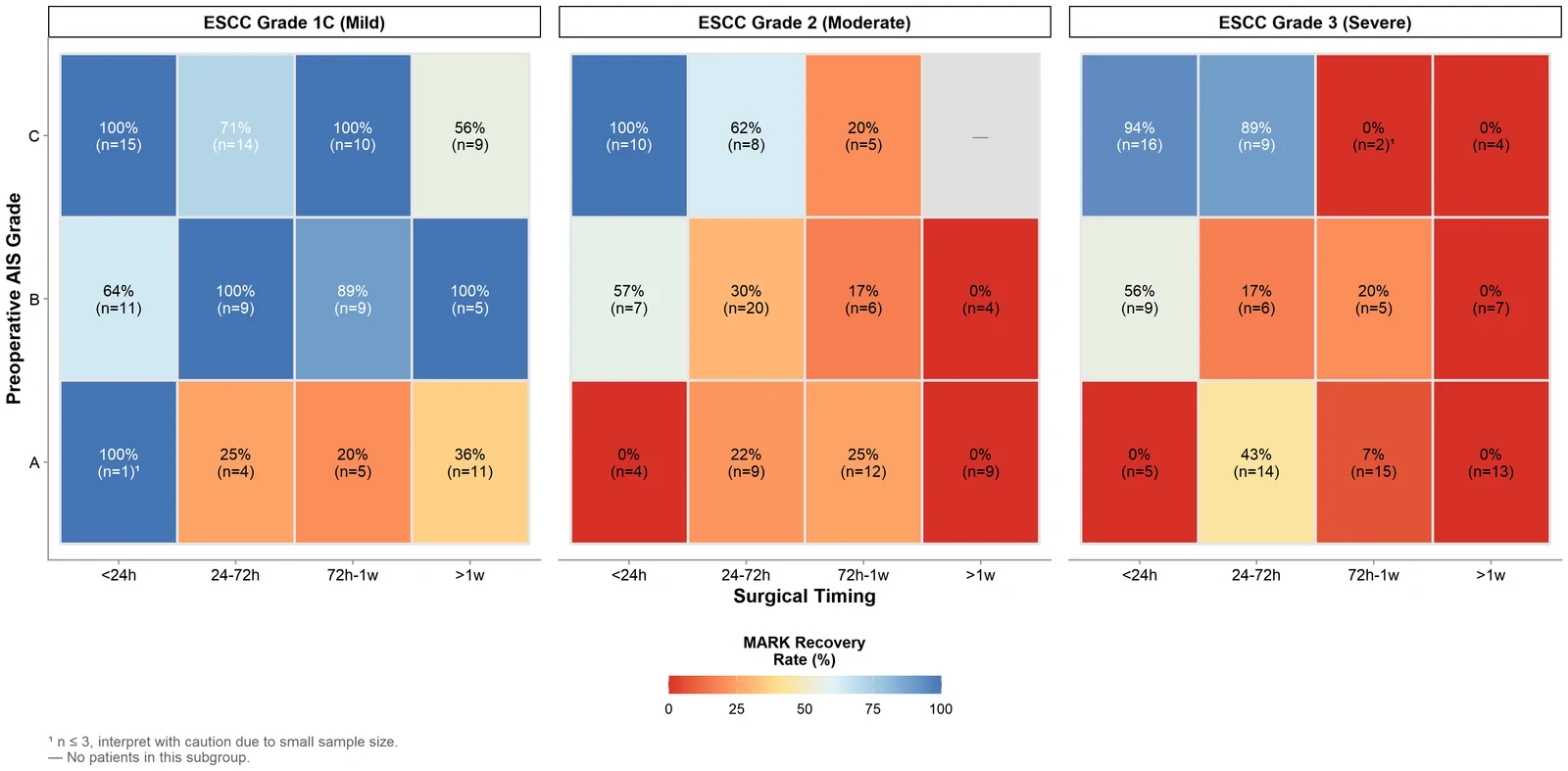
Neurological recovery rates by ESCC grade, preoperative AIS grade, and surgical timing. Heatmap showing three-way interaction on marked neurological recovery (AIS D/E). Each cell displays recovery rate (%) and sample size (n). Color intensity: blue = higher recovery, red = lower recovery. Highest recovery: ESCC 1C + AIS C + <24h (blue). Lowest: ESCC 3 + AIS A + >1w (red). ESCC, epidural spinal cord compression; AIS, American Spinal Injury Association Impairment Scale; h, hours; w, week.

Interaction among ESCC grade, AIS grade, and surgical timing. A heatmap was constructed to visualize the interaction of ESCC grade (1C, 2, 3), preoperative AIS grade (A, B, C), and surgical timing (<24h, 24–72h, 72h–1w, >1w) on marked neurological recovery (**Figure 6**). The color intensity represents the recovery rate, with blue indicating higher recovery rates and red indicating lower recovery rates. Each cell displays the recovery rate (%) and sample size (n).

The highest recovery rates were observed in patients with ESCC grade 1C, preoperative AIS C, and surgery within 24 hours (blue region). In contrast, the lowest recovery rates were observed in patients with ESCC grade 3, preoperative AIS A, and surgery delayed beyond 1 week (red region). Across all ESCC grades, early surgery (<24h) was consistently associated with better recovery compared to delayed surgery (>1w), particularly in patients with higher preoperative AIS grade (B/C). This exploratory heatmap suggests potential interactions among ESCC grade, AIS grade, and surgical timing. The interaction plot demonstrates that delayed surgery has a more pronounced negative impact on recovery in patients with severe cord compression (ESCC grade 3) and complete paralysis (AIS A), whereas patients with mild compression (ESCC grade 1C) and incomplete paralysis (AIS B/C) maintained relatively higher recovery rates even with moderate surgical delays. These findings highlight the critical importance of timely surgical intervention, especially in high-risk patient subgroups. Formal likelihood ratio tests confirmed significant pairwise interactions between ESCC grade and surgical timing (p = 0.003) and between preoperative AIS grade and surgical timing (p = 0.008); however, the full three-way interaction could not be reliably estimated owing to sparse data in several strata (**Supplementary Table S****-C4**). These findings should be interpreted as hypothesis-generating rather than confirmatory, and require validation in larger cohorts.

### Prediction model and validation

**Figure 7:**
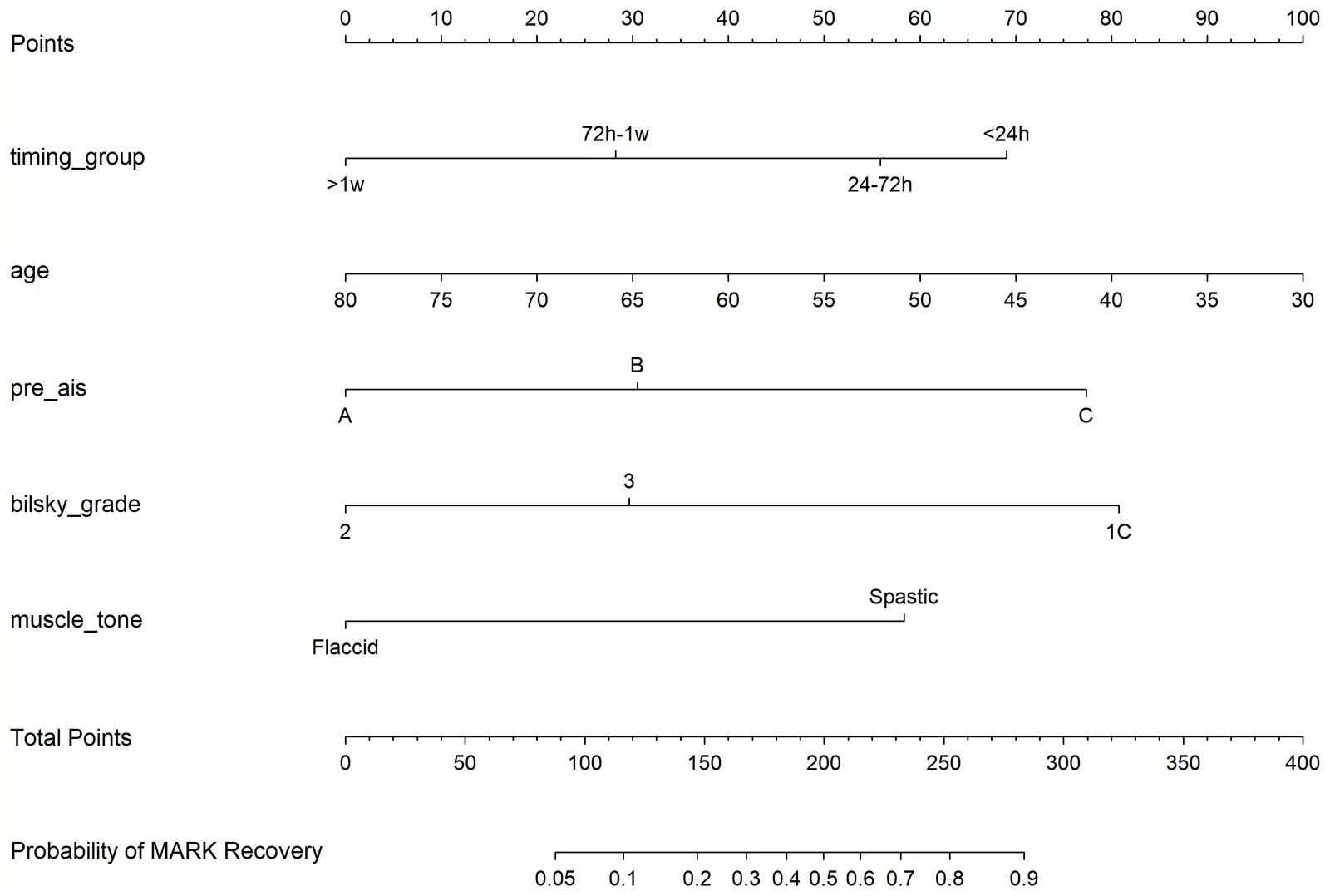
Nomogram for predicting marked neurological recovery (AIS D/E). The nomogram integrates five predictors: surgical timing, age, preoperative AIS grade, ESCC grade, and muscle tone. To use: locate each variable value, draw vertical line to “Points,” sum points, and read predicted probability from bottom axis. Bootstrap-corrected C-index = 0.873. AIS, American Spinal Injury Association Impairment Scale; ESCC, epidural spinal cord compression; h, hours; w, week.

Nomogram for predicting neurological recovery. A nomogram was developed based on the independent predictors identified in the multivariable Firth logistic regression model, including surgical timing, age, preoperative AIS grade, ESCC grade, and muscle tone (**Figure 7**). Each predictor was assigned a point value on the top scale. The total points sum corresponds to the predicted probability of achieving marked neurological recovery (AIS D/E) on the bottom scale. The nomogram demonstrated good discrimination with a bootstrap-corrected C-index of 0.873. Additional performance metrics were as follows: calibration slope (apparent: 1.000; bootstrap-corrected: 0.898), calibration intercept (apparent: 0.000; corrected: −0.006), and Brier score (apparent: 0.134; corrected: 0.147) (**Supplementary Table S****-C3**), indicating satisfactory model calibration and overall performance.

**Figure 8:**
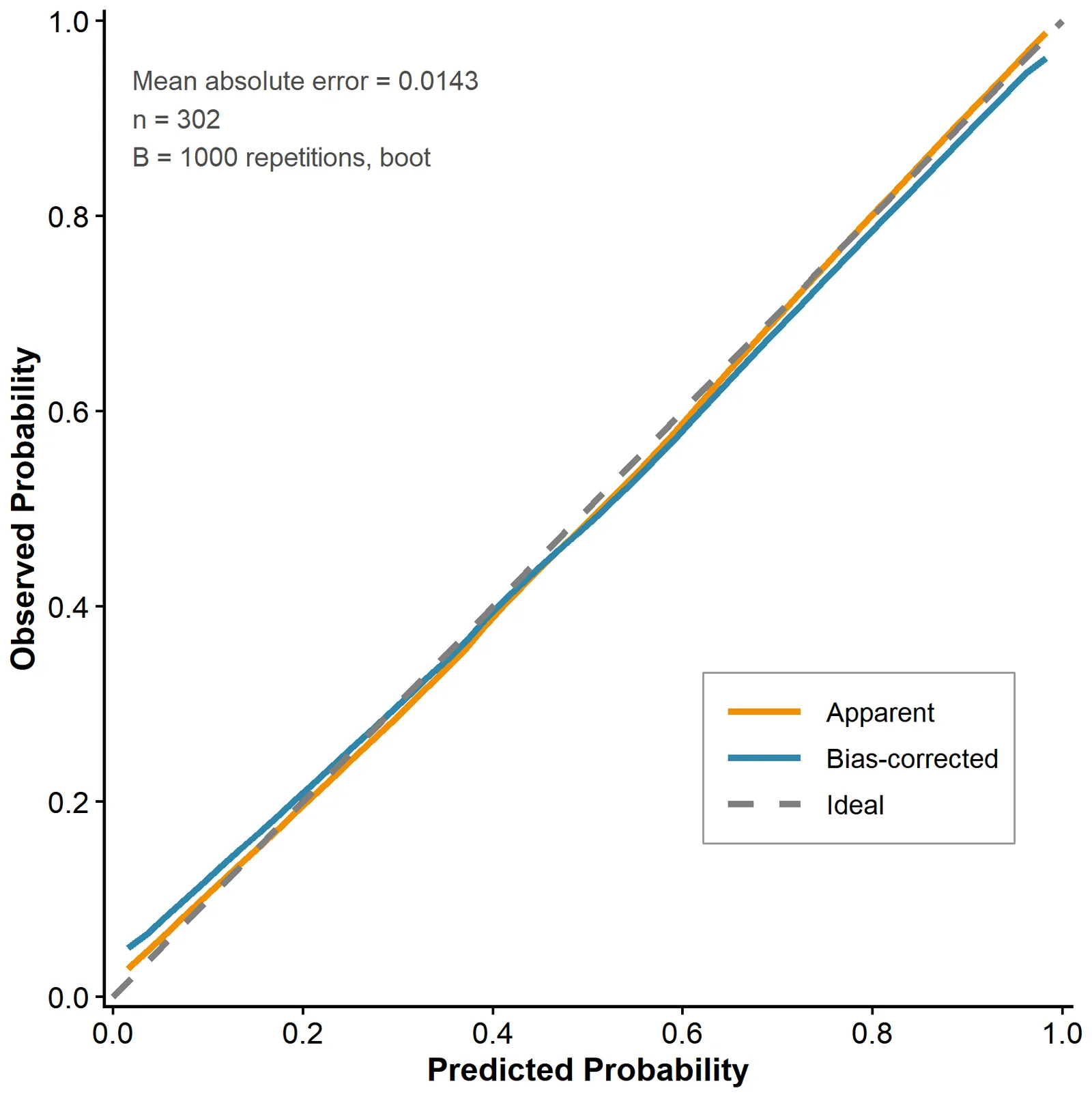
Calibration curve of the nomogram. Orange line = apparent curve; blue line = bias-corrected curve (1000 bootstrap resamples); gray dashed line = ideal calibration. The bias-corrected curve shows good agreement with the ideal line (MAE = 0.0143), indicating no significant overfitting. MAE, mean absolute error.

Model calibration. The calibration curve showed excellent agreement between the predicted probabilities and observed probabilities of marked neurological recovery (**Figure 8**). The bias-corrected curve (blue) closely followed the ideal diagonal line, with a mean absolute error (MAE) of 0.0143, indicating outstanding calibration performance of the nomogram.

**Figure 9:**
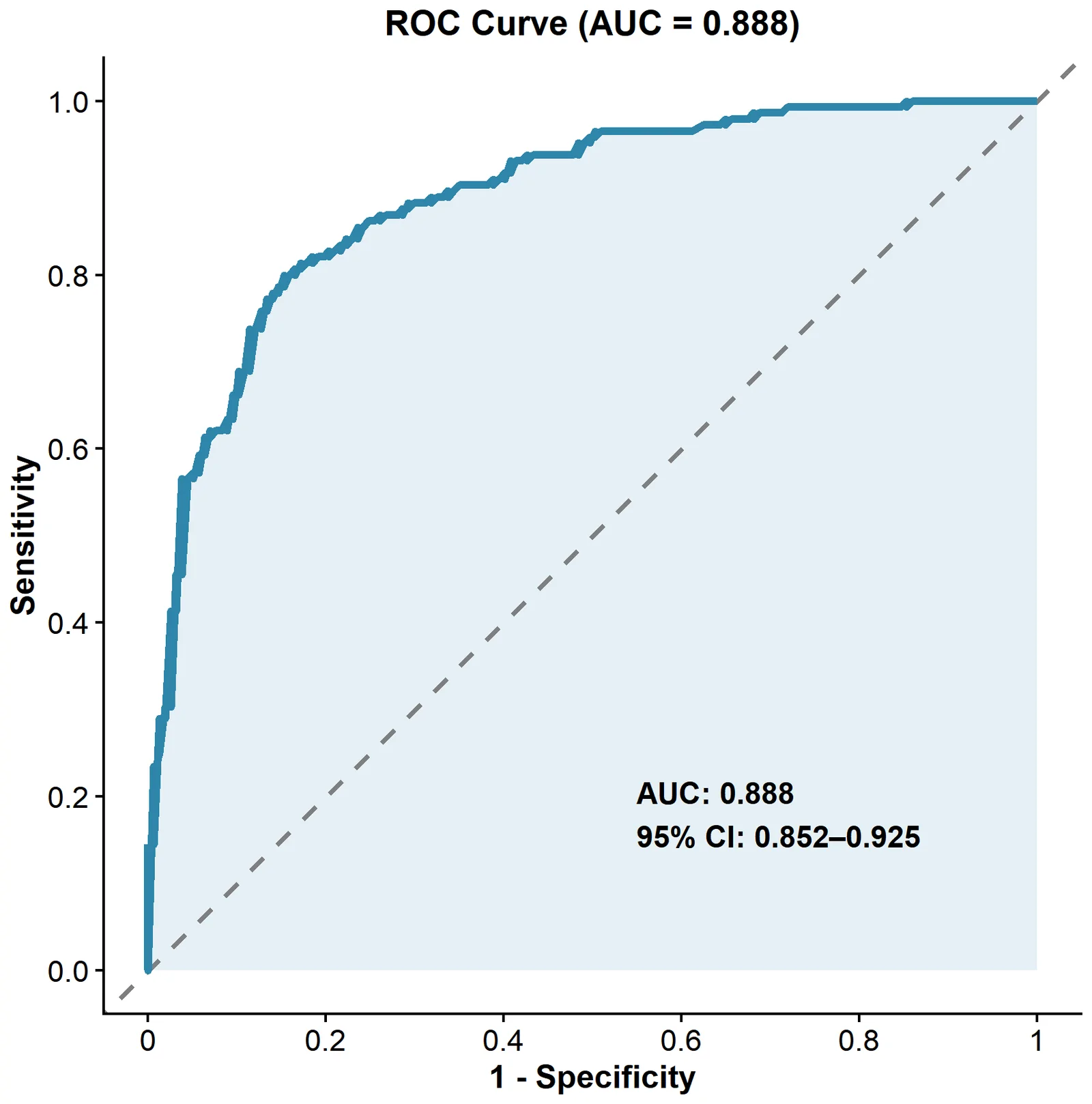
ROC curve of the nomogram. The AUC of 0.888 (95% CI: 0.852–0.925) indicates excellent discriminative ability. The blue curve deviates substantially from the diagonal reference line (AUC = 0.5), confirming the nomogram’s ability to distinguish patients likely to achieve marked neurological recovery. ROC, receiver operating characteristic; AUC, area under the curve; CI, confidence interval.

Discriminative ability. The receiver operating characteristic (ROC) curve demonstrated excellent discriminative ability of the nomogram, with an area under the curve (AUC) of 0.888 (95% CI: 0.852–0.925) (**Figure 9**). The blue curve deviated significantly from the diagonal reference line (AUC = 0.5), indicating that the nomogram can effectively distinguish patients who are likely to achieve marked neurological recovery from those who are not.

**Figure 10:**
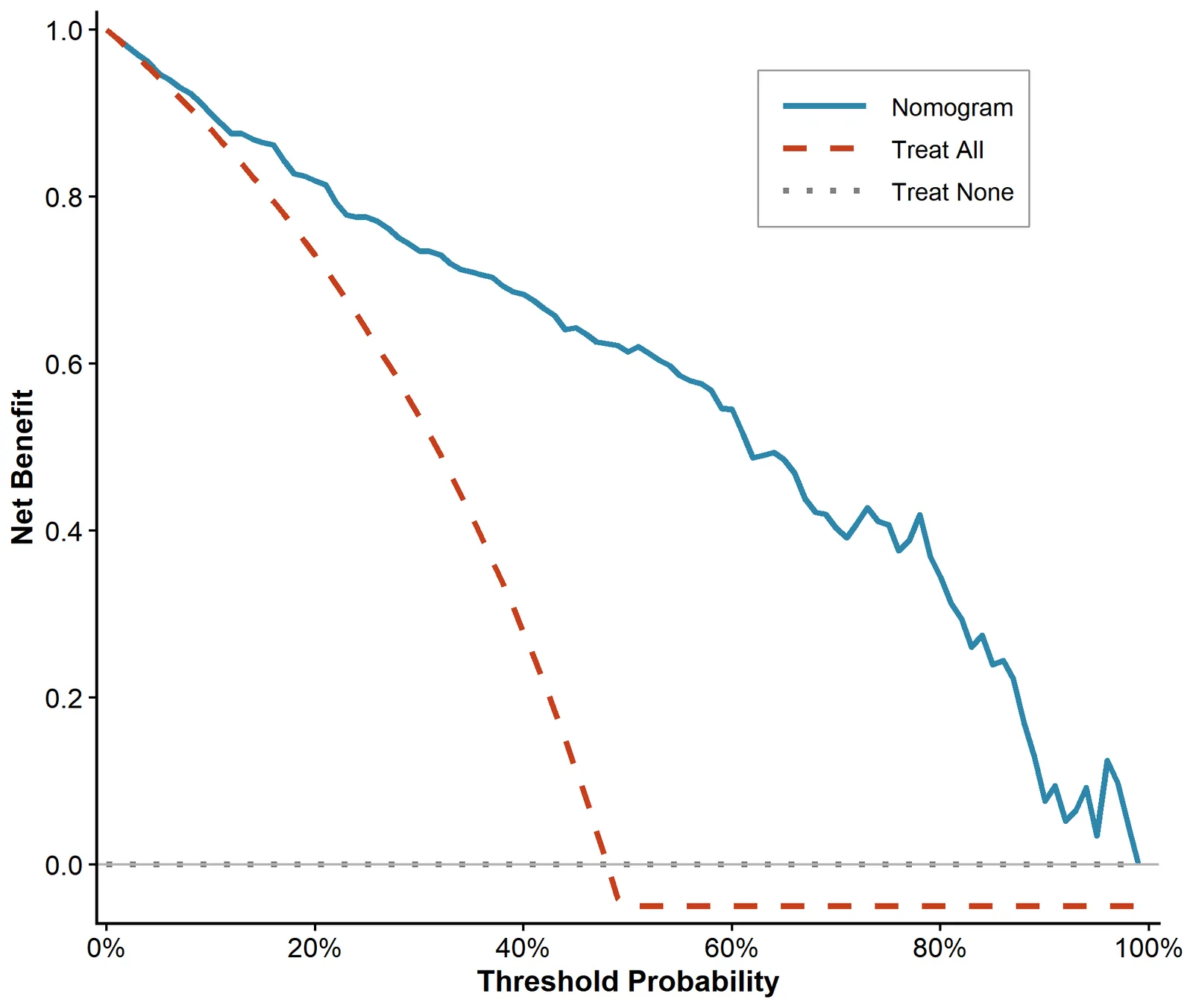
Decision curve analysis (DCA) of the nomogram. Blue solid line = nomogram; red dashed line = “treat all”; gray dotted line = “treat none.” The nomogram showed higher net benefit across a wide range of threshold probabilities (approximately 10–80%), supporting its clinical utility as a decision-support tool.

Clinical utility. Decision curve analysis (DCA) was performed to evaluate the clinical utility of the nomogram (**Figure 10**). The blue solid line represents the nomogram model, the red dashed line represents the “treat all” strategy, and the gray dotted line represents the “treat none” strategy. The nomogram curve showed higher net benefit compared to both the “treat all” and “treat none” strategies across a wide range of threshold probabilities (approximately 10–80%). Given that all patients in this cohort underwent surgery, this analysis should be interpreted as demonstrating the nomogram’s potential value for risk stratification within a surgical population, rather than as evidence of clinical decision-making utility in a broader treatment-selection context.

## Discussion

### Summary of main findings

In this retrospective cohort study of 302 patients with spinal metastasis-induced paralysis (AIS grades A–C), we found that earlier surgical intervention was significantly associated with better neurological recovery and longer overall survival. The 24-month survival probabilities were 69.2% for the <24h group, compared with only 19.4% for the >1w group (log-rank p < 0.001). Multivariable analysis confirmed that surgical timing, preoperative AIS grade, ESCC grade, and muscle tone were independent predictors of marked neurological recovery (AIS D/E). Notably, surgery within 72 hours to 1 week and beyond 1 week were associated with progressively worse outcomes, while the 24–72h group showed a non-significant trend toward worse recovery compared with the <24h group. The interaction between ESCC grade, AIS grade, and surgical timing was visualized using a heatmap, demonstrating that patients with severe cord compression (ESCC grade 3), complete paralysis (AIS A), and delayed surgery (>1w) had the poorest prognosis. Finally, we developed a nomogram incorporating five independent predictors, which showed excellent discrimination with a bootstrap-corrected C-index of 0.873 and an AUC of 0.888 (95% CI: 0.852–0.925). Calibration curve and decision curve analysis confirmed good model performance and clinical utility.

### Surgical timing and neurological recovery

MESCC is a neurosurgical emergency, and our findings support the critical role of early decompression in preserving neurological function (1–3, 12). Although the benefit of surgery is well-established, the optimal timing remains debated. Most studies have shown a progressive decline in neurological recovery with increasing delay. Fürstenberg et al. (6) reported improved recovery within 48 hours, Fan et al. (8) demonstrated superior ambulation in complete paralysis treated within 48 hours, and Chaichana et al. (7) identified <48-hour symptom duration as an independent predictor. Li et al. (9) further demonstrated meaningful neurological benefit when surgery was performed within one week, whereas Rades et al. (13) reported that treatment delay reduced neurological recovery. More recently, Quraishi et al. (14) and Van Tol et al. (15) further emphasized that delayed referral and surgical intervention are associated with inferior postoperative outcomes. Our data suggest that surgery within 24 hours yields maximal neurological benefit, with decreasing outcomes for longer delays. Mechanistically, prolonged compression may induce spinal cord ischemia, demyelination, and irreversible neuronal loss, supporting urgent decompression (11). Additional sensitivity analyses using alternative timing thresholds (48-hour cutoff) and continuous time modeling yielded consistent results (**Supplementary Tables S-A1**, **S-A2**, **S-B1**, **S-B2**).

However, it is important to acknowledge that the observed association between surgical timing and outcomes may be influenced by baseline neurological severity. Patients with delayed surgery had higher rates of complete paralysis, AIS A deficits, and flaccid muscle tone at presentation, which may reflect both more severe disease and barriers to timely referral. These baseline differences could have independently influenced both the timing of surgery and the likelihood of recovery. Despite multivariable adjustment for these factors, residual confounding by unmeasured variables—such as rate of neurological decline, time from symptom onset to diagnosis, and overall functional status—cannot be fully excluded in this retrospective design.

### Neurological predictors of recovery

Preoperative neurological status remained the strongest predictor of recovery (5, 7, 9). High-grade ESCC was associated with worse outcomes, consistent with prior validation of the Bilsky scale (3, 10). Interestingly, its effect was attenuated after adjusting for baseline neurological function and timing, indicating that severity primarily acts through preoperative neurological deficits. Muscle tone emerged as an additional independent predictor: flaccid paralysis was associated with poorer recovery and survival, reflecting more extensive spinal cord injury (11).

### Interpretation of severe deficits in ESCC grade 1C

The presence of AIS A–C deficits in patients with ESCC grade 1C—where imaging shows only spinal cord abutment without true compression—warrants discussion. Several mechanisms may explain this apparent paradox between imaging findings and clinical presentation. First, vascular compromise from epidural venous obstruction may cause cord ischemia and edema disproportionate to the degree of mechanical compression (16–18). Second, static supine MRI may not capture dynamic compression that occurs during physiological spinal motion, particularly in the presence of spinal instability (19). Third, the rapid progression characteristic of MESCC means that neurological deterioration may outpace imaging assessment, especially in patients with aggressive tumor biology (19, 20). Fourth, tumor-related inflammatory or paraneoplastic mechanisms may contribute to cord dysfunction independent of direct mechanical compression (21).

Conversely, it is equally noteworthy that some patients with high-grade cord compression (ESCC grade 3) may present with relatively preserved neurological function. This inverse discordance—severe imaging findings without corresponding clinical deficits—is also observed in clinical practice and may reflect individual differences in spinal cord reserve capacity (22), the chronicity of compression allowing gradual neural adaptation (23, 24), or variations in collateral vascular supply that maintain cord perfusion despite mechanical deformation (24). Although these mechanisms have been primarily studied in degenerative cervical myelopathy, similar principles may apply to metastatic compression, particularly in cases of indolent tumor growth.

Taken together, these bidirectional discrepancies between radiological and clinical findings underscore the complexity of MESCC pathophysiology and highlight that imaging severity alone is insufficient for predicting neurological status. We acknowledge that these proposed mechanisms are speculative and cannot be directly tested using our retrospective dataset. Further prospective studies incorporating dynamic imaging, vascular assessment, and biomarkers of inflammation or neural injury are needed to elucidate the true pathophysiology underlying the variable relationship between cord compression severity and neurological deficits. We hope to address this question in future work.

### Confounding by indication and selection bias

An important limitation of this study is the potential for confounding by indication. Patients who underwent earlier surgery may have differed systematically from those with delayed surgery in ways that are difficult to fully capture. For example, patients presenting with rapid neurological decline may have been prioritized for urgent surgery, while those with more indolent presentations or logistical barriers may have experienced delays. Similarly, patients with severe baseline deficits (AIS A, flaccid paralysis) may have been perceived as having poorer prognosis, potentially influencing clinical decision-making and referral patterns. Although we adjusted for key baseline variables including AIS grade, ESCC grade, and muscle tone, residual confounding remains a concern. The observed associations should therefore be interpreted as hypothesis-generating rather than definitive evidence of causality.

### Survival implications of neurological recovery

Neurological recovery strongly correlated with survival. Patients who regained function lived longer than those who did not, supporting findings from Li et al. (9) and rehabilitation studies (25). Recovery likely mitigates complications of immobility, facilitates rehabilitation, and enables systemic therapy. Our multivariable analysis confirmed surgical timing, ESCC grade, muscle tone, age, and sex as independent prognostic factors. Traditional scores (Tokuhashi, Tomita) focus on tumor biology and systemic disease (26, 27), but functional and neurological factors also play key roles (28–30). Neurological improvement may further improve quality of life (4, 31).

### Oncological factors and survival interpretation

It is essential to acknowledge that survival outcomes in metastatic spinal disease are influenced by a complex interplay of factors beyond surgical timing, including primary tumor histology, systemic disease burden, presence of visceral metastases, performance status, and access to effective systemic therapies. In our cohort, lung cancer was the most common primary tumor (49.3%), which is typically associated with poorer prognosis compared to breast or prostate cancer. Although we did not include tumor type in the multivariable survival model due to sample size constraints across tumor categories, this factor likely contributed to the observed mortality rates. Future studies with larger cohorts should incorporate tumor-specific analyses to better delineate the independent contribution of surgical timing to survival across different malignancy types. In a sensitivity analysis additionally adjusting for postoperative systemic anticancer treatment, the surgical timing effect remained directionally consistent (**Supplementary Table S-C2**).The unexpected finding that female sex was independently associated with increased mortality risk (HR = 1.40, p = 0.044) warrants careful interpretation. This association is unlikely to reflect a direct biological effect of sex on survival. Instead, it may be confounded by differences in tumor biology, as breast cancer—the second most common primary tumor in our cohort (14.6%)—predominantly affects women and may have distinct metastatic behavior and treatment response patterns. Additionally, differences in access to healthcare, timing of presentation, or receipt of systemic therapies may vary by sex. This finding should be considered exploratory and requires validation in larger, more diverse cohorts with detailed information on systemic treatment history.

### Nomogram and clinical implications

We developed a nomogram to predict neurological recovery, expanding upon prior work by Li et al. (9) by integrating AIS grade, ESCC grade, muscle tone, and surgical timing. The model demonstrated excellent discrimination (C-index 0.873, AUC 0.888) and calibration, and decision curve analysis confirmed its clinical utility (32). However, this nomogram should be considered a preliminary prognostic tool for risk stratification rather than a validated clinical decision aid. In particular, because all patients in this cohort underwent surgical decompression, the decision curve analysis cannot be used to define a threshold for treatment selection between surgical and non-surgical management. The net benefit demonstrated by the DCA reflects the nomogram’s ability to identify patients more or less likely to achieve marked neurological recovery within a uniformly operated cohort, and should not be extrapolated to guide surgery-versus-no-surgery decisions. External validation in independent, geographically diverse cohorts is essential before it can be reliably used for patient counseling or surgical decision-making in routine clinical practice. Until such validation is performed, the nomogram may serve primarily as a framework for understanding the relative contribution of different prognostic factors to neurological recovery.

Strengths include a large contemporary cohort, comprehensive multivariable analysis, and the use of Firth penalized logistic regression to reduce small-sample bias and address separation (33, 34). Limitations include the retrospective design, single-center setting, and need for external validation before widespread clinical implementation. Furthermore, our survival analysis was limited by the lack of detailed oncological data, including performance status, systemic disease burden, number and location of visceral metastases, primary tumor biology, systemic therapy regimens, postoperative radiotherapy, steroid use, and treatment line. These variables are known to significantly influence survival in patients with metastatic spinal disease. Their absence in our dataset limits our ability to fully adjust for oncological prognostic factors, and the observed survival benefits associated with early surgery should therefore be interpreted with caution. The association may be partly confounded by these unmeasured factors. Additionally, the inclusion of patients with ESCC grade 1C—who present with severe neurological deficits despite the absence of true radiographic cord compression—introduces interpretive complexity. A sensitivity analysis restricted to ESCC grade 2–3 patients (n = 199) yielded results consistent with the primary analysis, supporting the robustness of the main findings in the presence of confirmed cord compression (**Supplementary Tables S-D1**, **S-D2**).

The definition of paralysis onset was determined retrospectively from medical records, which may be subject to recall bias, particularly in patients with gradual neurological decline or delayed referral. The exact timing of symptom onset may be less reliable in late-presenting cases, and this limitation should be considered when interpreting the association between surgical timing and outcomes. Furthermore, although neurological function was assessed at standardized postoperative time points (at discharge, and at 1, 3, and 6 months), discrete time-point AIS values were not extracted as a separate variable in our retrospective database; the primary neurological outcome therefore reflects the best assessment at last available follow-up, with LOCF applied to patients with early mortality. A sensitivity analysis excluding the 25 patients who died within 3 months of surgery (n = 277) yielded results virtually identical to the primary analysis, suggesting that LOCF introduced negligible bias at the 3-month timeframe. A fixed 6-month analysis was not performed, as the high early mortality rate in the delayed-surgery groups (>1w: 54.8% died before 6 months) would introduce severe survivorship bias. Future prospective studies should prospectively record AIS grades at fixed postoperative time points to enable more rigorous time-specific analyses.

Because all patients in this cohort underwent surgery, the decision curve analysis primarily illustrates the potential added value of the nomogram for risk stratification rather than defining a clear treatment threshold. Future studies including both surgical and non-surgical cohorts are needed to validate the nomogram’s utility for treatment selection. The heatmap-based interaction analysis is exploratory. Formal testing of the three-way interaction (ESCC grade × AIS grade × surgical timing) was not feasible due to sparse data (several cells n ≤ 3), and the observed patterns should therefore be considered hypothesis-generating. Larger multicenter cohorts are needed to confirm these interaction effects.

Importantly, reducing delays in diagnosis, referral, and surgical treatment should remain a clinical priority to optimize outcomes in this vulnerable patient population. Recent evidence suggests that implementation of structured multidisciplinary diagnostic-therapeutic algorithms involving emergency physicians, oncologists, spine surgeons, neuroradiologists, and radiation oncologists can significantly reduce time to treatment and improve outcomes in patients with vertebral metastases and spinal cord compression (35). Such organizational interventions may be as important as surgical technique in improving patient outcomes, and future research should evaluate the effectiveness of standardized care pathways in reducing avoidable delays. The findings of the present study, identifying delayed surgery as a negative prognostic factor, provide further rationale for the development and implementation of rapid-response protocols for MESCC.

## Conclusion

In conclusion, early surgical decompression—ideally within 24 hours—is associated with superior neurological recovery in patients with spinal metastasis-induced paralysis. Delayed surgery was also associated with increased mortality risk; however, as key oncological variables including performance status, systemic disease burden, and visceral metastases were unavailable in this retrospective cohort, these survival findings should be interpreted as associations rather than causal effects. Surgery performed within one week was associated with meaningful neurological benefits compared with further delays, providing a practical clinical window for intervention. Preoperative AIS grade, ESCC grade, and muscle tone are independent prognostic factors that should be incorporated into preoperative risk stratification. The developed nomogram offers a preliminary tool for individualized prediction of marked neurological recovery and, pending external validation, may facilitate clinical decision-making in patients with MESCC-associated paralysis.

Future prospective multicenter studies are warranted to externally validate the nomogram and assess the long-term durability of neurological recovery and survival benefits, particularly in patients with indolent tumor types. Incorporation of quality-of-life measures, patient-reported outcomes, frailty indices, and detailed systemic therapy data may further improve risk stratification and optimize treatment planning. Comparative effectiveness studies evaluating surgery versus contemporary radiotherapy approaches, including stereotactic body radiotherapy, in carefully selected patient subgroups are also needed. Finally, reducing delays in diagnosis, referral, and surgical treatment should remain a clinical priority to optimize outcomes in this vulnerable patient population.

## Data Availability

The raw data supporting the conclusions of this article will be made available by the authors, without undue reservation.
