# Supplementary material for: Surgical timing, neurological recovery, and survival in spinal metastasis-induced paralysis: a retrospective cohort study with nomogram development
Source: Front Oncol. 2026 Sep 7;16:1901644. doi: 10.3389/fonc.2026.1901644 (PMC13590400; doi:10.3389/fonc.2026.1901644)
Supplement: Supplementary file 1 [file Table1.docx]

Supplementary Material

# 1 Supplementary Figures and Tables

Supplementary Table. Sensitivity Analysis: Firth Penalized Logistic Regression for Marked Neurological Recovery

| **Variable** | **Primary OR (95% CI)** | **Primary p-value** | **Sens-1 OR (95% CI)** | **Sens-1 p-value** | **Sens-2 OR (95% CI)** | **Sens-2 p-value** |
| --- | --- | --- | --- | --- | --- | --- |
| Timing: 24-72h vs <24h | 0.69 (0.30–1.54) | 0.361 | 0.68 (0.30–1.54) | 0.357 | 0.66 (0.29–1.49) | 0.317 |
| Timing: 72h-1w vs <24h | 0.34 (0.13–0.84) | 0.019* | 0.34 (0.13–0.84) | 0.019* | 0.37 (0.14–0.94) | 0.037* |
| Timing: >1w vs <24h | 0.17 (0.06–0.48) | <0.001*** | 0.17 (0.06–0.49) | <0.001*** | 0.23 (0.07–0.67) | 0.007** |
| Age (per year) | 0.95 (0.92–0.98) | 0.002** | 0.95 (0.92–0.98) | 0.002** | 0.95 (0.92–0.98) | 0.004** |
| Sex (Female vs Male) | 0.76 (0.40–1.40) | 0.378 | 0.77 (0.41–1.43) | 0.403 | 0.84 (0.44–1.59) | 0.595 |
| Preop AIS B vs A | 2.14 (1.01–4.60) | 0.048* | 2.10 (0.99–4.53) | 0.054 | 1.79 (0.83–3.91) | 0.135 |
| Preop AIS C vs A | 6.86 (3.17–15.49) | <0.001*** | 6.72 (3.10–15.20) | <0.001*** | 6.49 (2.90–15.17) | <0.001*** |
| ESCC grade 2 vs 1C | 0.14 (0.06–0.29) | <0.001*** | 0.13 (0.06–0.29) | <0.001*** | 0.13 (0.06–0.28) | <0.001*** |
| ESCC grade 3 vs 1C | 0.28 (0.12–0.62) | 0.002** | 0.28 (0.12–0.62) | 0.002** | 0.29 (0.12–0.66) | 0.003** |
| Muscle tone (Flaccid vs Spastic) | 0.23 (0.12–0.45) | <0.001*** | 0.24 (0.12–0.46) | <0.001*** | 0.24 (0.12–0.46) | <0.001*** |

Abbreviations: OR, odds ratio; CI, confidence interval; AIS, ASIA Impairment Scale; ESCC, epidural spinal cord compression. *p<0.05; **p<0.01; ***p<0.001. Shaded rows: key predictors.

In this cohort, no patients died within 1 month of surgery (minimum survival: 2 months); all patients received at least one postoperative neurological assessment at hospital discharge. Primary analysis: all 302 patients (n = 0 died before first postoperative assessment). Sensitivity 1 excludes 2 patient(s) who died within 2 months. Sensitivity 2 excludes 25 patients who died within 3 months. Results were consistent across all analyses, supporting the robustness of the primary findings.

**Table S-A0. Recovery rates by new timing categories**

| **timing_group2** | **N** | **Recovered** | **Rate** |
| --- | --- | --- | --- |
| <24h | 78 | 57 | 73.1% |
| 24-48h | 31 | 22 | 71% |
| 48-72h | 62 | 26 | 41.9% |
| >72h | 131 | 40 | 30.5% |

**Table S-A1. Firth logistic regression — new timing categories (<24h / 24-48h / 48-72h / >72h)**

| **Variable** | **OR (95% CI)** | **p-value** |
| --- | --- | --- |
| Timing: 24-48h vs <24h | 0.59 (0.19–1.87) | 0.366 |
| Timing: 48-72h vs <24h | 0.74 (0.30–1.79) | 0.497 |
| Timing: >72h vs <24h | 0.26 (0.11–0.60) | 0.002** |
| Age (per year) | 0.95 (0.92–0.98) | 0.002** |
| Sex (Female vs Male) | 0.74 (0.40–1.38) | 0.349 |
| Preop AIS B vs A | 2.17 (1.03–4.68) | 0.042* |
| Preop AIS C vs A | 7.07 (3.22–16.22) | <0.001*** |
| ESCC grade 2 vs 1C | 0.15 (0.07–0.32) | <0.001*** |
| ESCC grade 3 vs 1C | 0.30 (0.13–0.65) | 0.002** |
| Muscle tone (Flaccid vs Spastic) | 0.22 (0.11–0.42) | <0.001*** |

Reference group: <24h. Shaded rows = timing variables. OR for marked neurological recovery (AIS D/E).

**Table S-A2. Cox regression — new timing categories (<24h / 24-48h / 48-72h / >72h)**

| **Variable** | **HR (95% CI)** | **p-value** |
| --- | --- | --- |
| Timing: 24-48h vs <24h | 1.04 (0.44–2.44) | 0.935 |
| Timing: 48-72h vs <24h | 1.66 (0.97–2.82) | 0.064 |
| Timing: >72h vs <24h | 2.66 (1.61–4.38) | <0.001*** |
| Age (per year) | 1.03 (1.01–1.05) | 0.004** |
| Sex (Female vs Male) | 1.39 (1.00–1.93) | 0.047* |
| Preop AIS B vs A | 0.69 (0.48–1.01) | 0.059 |
| Preop AIS C vs A | 0.59 (0.37–0.93) | 0.025* |
| ESCC grade 2 vs 1C | 1.26 (0.82–1.94) | 0.298 |
| ESCC grade 3 vs 1C | 1.34 (0.87–2.07) | 0.180 |
| Muscle tone (Flaccid vs Spastic) | 2.42 (1.66–3.52) | <0.001*** |

Reference group: <24h. Shaded rows = timing variables. HR for overall mortality.

**Table S-B1. Firth logistic regression — surgical timing as continuous variable (log2-hours)**

| **Variable** | **OR (95% CI)** | **p-value** |
| --- | --- | --- |
| Surgical timing (per doubling of hours)* | 0.63 (0.48–0.82) | <0.001*** |
| Age (per year) | 0.95 (0.92–0.99) | 0.004** |
| Sex (Female vs Male) | 0.77 (0.41–1.42) | 0.407 |
| Preop AIS B vs A | 2.16 (1.02–4.64) | 0.044* |
| Preop AIS C vs A | 6.68 (3.08–15.03) | <0.001*** |
| ESCC grade 2 vs 1C | 0.14 (0.06–0.31) | <0.001*** |
| ESCC grade 3 vs 1C | 0.28 (0.13–0.63) | 0.002** |
| Muscle tone (Flaccid vs Spastic) | 0.25 (0.12–0.49) | <0.001*** |

* OR per doubling of hours to surgery (e.g., 12h to 24h, or 24h to 48h).

**Table S-B2. Cox regression — surgical timing as continuous variable (log2-hours)**

| **Variable** | **HR (95% CI)** | **p-value** |
| --- | --- | --- |
| Surgical timing (per doubling of hours)* | 1.51 (1.29–1.75) | <0.001*** |
| Age (per year) | 1.03 (1.01–1.04) | 0.005** |
| Sex (Female vs Male) | 1.44 (1.04–2.00) | 0.027* |
| Preop AIS B vs A | 0.68 (0.47–0.99) | 0.043* |
| Preop AIS C vs A | 0.61 (0.39–0.97) | 0.036* |
| ESCC grade 2 vs 1C | 1.34 (0.87–2.08) | 0.185 |
| ESCC grade 3 vs 1C | 1.49 (0.97–2.30) | 0.072 |
| Muscle tone (Flaccid vs Spastic) | 2.12 (1.44–3.10) | <0.001*** |

* HR per doubling of hours to surgery.

Abbreviations: OR, odds ratio; HR, hazard ratio; CI, confidence interval; AIS, ASIA Impairment Scale; ESCC, epidural spinal cord compression. *p<0.05; **p<0.01; ***p<0.001.

**Table S-C1. Preoperative AIS grade distribution among patients with ESCC grade 1C**

| **Preoperative AIS grade** | **n** | **% within ESCC grade 1C** |
| --- | --- | --- |
| A (complete paralysis) | 21 | 20.4% |
| B | 34 | 33.0% |
| C | 48 | 46.6% |
| Total | 103 | 100% |

Note: Among the 103 patients with ESCC grade 1C, 26 (25.2%) had complete paralysis (paralysis_type) and 77 (74.8%) had incomplete paralysis. ESCC grade 1C is defined as thecal sac deformation with spinal cord abutment but without radiographically evident cord compression; see Discussion for interpretation of severe deficits occurring in this subgroup.

**Table S-C2. Sensitivity analysis: Cox proportional hazards model additionally adjusted for postoperative systemic anticancer treatment**

| **Variable** | **HR (95% CI)** | **p-value** |
| --- | --- | --- |
| Timing: 24-72h vs <24h | 1.65 (0.99-2.75) | 0.056 |
| Timing: 72h-1w vs <24h | 1.77 (1.03-3.03) | 0.039* |
| Timing: >1w vs <24h | 4.74 (2.73-8.23) | <0.001*** |
| Age (per year) | 1.02 (1.00-1.04) | 0.026* |
| Sex (Female vs Male) | 1.31 (0.94-1.83) | 0.105 |
| Preop AIS B vs A | 1.00 (0.68-1.46) | 0.988 |
| Preop AIS C vs A | 0.88 (0.56-1.39) | 0.594 |
| ESCC grade 2 vs 1C | 1.32 (0.84-2.06) | 0.230 |
| ESCC grade 3 vs 1C | 1.20 (0.78-1.86) | 0.406 |
| Muscle tone (Flaccid vs Spastic) | 1.27 (0.87-1.85) | 0.215 |
| Postoperative systemic treatment (Yes vs No) | 0.09 (0.05-0.16) | <0.001*** |

Note: Reference groups: Timing <24h, ESCC 1C, Preop AIS A, Muscle tone spastic, Sex male, no postoperative systemic treatment. Postoperative systemic anticancer treatment was the only additional cancer-related treatment variable systematically available in this retrospective cohort. The surgical timing effect remained directionally consistent after this adjustment. *p<0.05; ***p<0.001.

**Table S-C3. Bootstrap internal validation of the nomogram (1000 repetitions): discrimination and calibration metrics**

| **Metric** | **Apparent** | **Bootstrap-corrected** |
| --- | --- | --- |
| C-index | 0.888 | 0.873 |
| Calibration slope | 1.000 | 0.898 |
| Calibration intercept | 0.000 | -0.006 |
| Brier score | 0.134 | 0.147 |

Note: Calibration slope and intercept ideal values are 1 and 0, respectively. Internal validation was performed using bootstrap resampling in which the entire model-fitting process was repeated within each bootstrap sample (rms package), providing optimism-corrected estimates in accordance with TRIPOD recommendations.

**Table S-C4. Formal likelihood ratio tests for pairwise and three-way interactions in the logistic regression model for marked neurological recovery**

| **Interaction term** | **LRT Chi-square (df)** | **p-value** |
| --- | --- | --- |
| ESCC grade x Preoperative AIS grade | 3.07 (4) | 0.546 |
| ESCC grade x Surgical timing | 19.74 (6) | 0.003** |
| Preoperative AIS grade x Surgical timing | 17.40 (6) | 0.008** |
| ESCC grade x AIS grade x Surgical timing (3-way) | N/A (model non-convergence) | Not estimable (sparse data) |

Note: All models adjusted for surgical timing, age, sex, preoperative AIS grade, ESCC grade, and muscle tone (main effects) plus the indicated interaction term(s). The full three-way interaction term (corresponding to the heatmap in Figure 5) could not be reliably estimated owing to sparse data in several strata (some cells n<=3) and should be interpreted as exploratory. **p<0.01.
